# SHP-2-Mediated Upregulation of ZEB1 Is Important for PDGF-B-Induced Cell Proliferation and Metastatic Phenotype in Triple Negative Breast Cancer

**DOI:** 10.3389/fonc.2020.01230

**Published:** 2020-08-06

**Authors:** Lei Zhang, Chenwei Yuan, Jing Peng, Liheng Zhou, Yiwei Jiang, Yanping Lin, Wenjin Yin, Shuguang Xu, Jun Ma, Jinsong Lu

**Affiliations:** ^1^Department of Breast Surgery, School of Medicine, Renji Hospital, Shanghai Jiao Tong University, Shanghai, China; ^2^Department of Translational Skin Cancer Research, University Hospital Essen, Essen, Germany; ^3^Eye Institute, Eye & ENT Hospital, Shanghai Medical College, Fudan University, Shanghai, China

**Keywords:** PDGF-B, SHP-2, ZEB1, TNBC, metastasis

## Abstract

**Background:** Triple negative breast cancer (TNBC), a fatal malignant tumor, is characterized by a lack of estrogen and progesterone hormone receptors and overexpression of HER2. Due to its characteristics, there are no effective targeted therapies for TNBC. Therefore, it is critical to identify the crucial factors that participate in modulating TNBC progression and explore the underlying molecular mechanism.

**Methods:** CCK-8, bromodeoxyuridine incorporation, western blotting, qPCR, and transwell assays were utilized to evaluate breast cancer cell proliferation, migration, and invasion.

**Results:** Activation of platelet-derived growth factor (PDGF)-B/PDGF receptor (PDGFR) promoted the proliferation and metastatic phenotype of TNBC cells; however, these effects were attenuated by SHP-2 knockdown. Moreover, PDGF-B promoted the expression of zinc finger E-box binding homeobox 1 (ZEB1) by downregulating the expression of miR-200. Furthermore, knockdown of ZEB1 mitigated the promoting effects of PDGF-B on cell proliferation and migration. In addition, the regulatory effects of PDGF-B on miR-200 and ZEB1 were mediated through the SHP-2/Akt pathway.

**Conclusion:** Our findings highlight the important roles of PDGF-B/PDGFR and their downstream signaling pathways in regulating cell proliferation and metastatic phenotype in TNBC. Hence, these molecules may serve as novel therapeutic targets for TNBC in the future.

## Introduction

Breast cancer is one of the most common tumors among women worldwide. In 2017, breast cancer represented almost 30% of newly diagnosed carcinomas in women (1). Breast cancer is also the leading cause of cancer-related death in young Chinese women (2). The majority of cancer-induced deaths are caused by distant metastasis, although the rates of metastasis and mortality have decreased in patients with breast cancer (3). Certain complex mechanisms, such as epithelial-mesenchymal transition (EMT) and angiogenesis are involved in the regulation of distant metastasis (4–6). Therefore, to identify novel potential therapeutic targets for breast cancer, the mechanisms underlying uncontrolled growth and distant metastasis in breast cancer need to be further characterized.

Zinc finger E-box binding homeobox 1 (ZEB1), a transcription factor, can bind to E-box (5′-CACGTG-3′) DNA sequences and regulate the expression of several target genes (7). Reports have shown that ZEB1 acts as a transcriptional repressor (8, 9) or activator (10) in various cancer types. EMT-related genes, such as E-cadherin are the most well-known targets of ZEB1 (11); thus, many researchers have focused on the role of ZEB1 in migration and invasion in various cancers (12, 13). Accumulating evidence indicates that ZEB1 acts as a critical regulator of cancer cell proliferation and apoptosis (14, 15). However, the exact regulatory mechanisms of ZEB1 in breast cancer cell proliferation and invasion remain to be elucidated.

Platelet-derived growth factor (PDGF), identified in the late 1970s, is the serum component that is responsible for the proliferation of cells of mesenchymal origin (16). The PDGF family consists of four different ligands encoded by different genes, and there are five homo-/hetero-dimeric forms of PDGF ligands (PDGF-AA, PDGF-AB, PDGF-BB, PDGF-CC, and PDGF-DD). All PDGF dimers interact with the receptor tyrosine kinases, PDGFRα and PDGFRβ (17). Moreover, overexpression of both PDGF and the PDGF receptor (PDGFR) has been reported in several human tumor types, including gastric, colon, pancreatic, lung, and ovarian carcinomas and glioblastomas (18, 19). Although previous reports have indicated that PDGF-B plays a paracrine role in malignant and benign breast epithelial cell proliferation and lymphatic metastasis (20, 21), the mechanism by which PDGF-B/PDGFR signaling mediates breast cancer progression is poorly understood.

In this study, we aimed to investigate the relationship between PDGF-B/PDGFR signaling and ZEB1 in breast cancer cell proliferation and metastatic phenotype, as well as explore the underlying mechanisms. Our results showed that exogenous PDGF-B promoted triple negative breast cancer (TNBC) cell proliferation and metastatic phenotype, but treatment with a PDGFR inhibitor (imatinib) or siRNA attenuated these effects. Next, we examined the underlying mechanism and found that the miR-200**-**ZEB1 feedback loop mediated by PDGF-B-induced SHP-2 plays an important role in TNBC development.

## Materials and Methods

### Cell Culture and Treatments

MDA-MB-231 and MDA-MB-436 human TNBC cell lines were obtained from ATCC (Manassas, USA). Cells were cultured in Dulbecco's modified Eagle's medium containing 10% fetal bovine serum (FBS) and maintained in a 37°C incubator with an atmosphere containing 5% CO_2_. To avoid the interference of growth factors present in FBS, all experiments were performed using serum-free media. The culture medium was replaced with serum-free medium before each experiment. All cells were starved for 16 h, and then treated with 5 μM imatinib or exogenous PDGF-BB (20 ng/mL) in serum-free medium as indicated. The treatment time was 48 h for Cell Counting Kit-8 (CCK-8) and bromodeoxyuridine (BrdU) incorporation assays, and 24 h for other assays.

### CCK-8 Assay

The CCK-8 assay (CCK-8, Dojindo) was used to determine cell viability. Two thousand cells per well were cultured in 96-well culture plates at 37°C in 5% CO_2_. Next, 10 μL of CCK-8 solution was added to each well and incubated for 2 h at 37°C. The absorbance at 450 nm was then measured using a microplate reader.

### BrdU Incorporation Assay

The BrdU incorporation assay was performed as previously described (22). In brief, 5,000 cells per well were seeded in 96-well microplates and allowed to attach overnight. The incorporation of BrdU into newly synthesized DNA was then examined with a cell proliferation ELISA kit (Roche), according to the manufacturer's instructions. A microplate reader was used to determine the optical density of each well at 450/595 nm.

### SHP2 Activity Assay

SHP2 phosphatase activity was determined using the human/mouse/rat active DuoSet IC kit (R&D Systems; DYC3790-2). MDA-MB-231 and MDA-MB-436 cells were treated with 5 μM imatinib or 20 ng/mL PDGF-BB for 24 h, rinsed two times with TBS (25 mM Tris, 150 mM NaCl, pH 7.5), and solubilized in lysis buffer (50 mM HEPES, 0.1 mM EDTA, 0.1 mM EGTA, 0.5% NP-40 Alternative, 120 mM NaCl, 25 μg/mL Leupeptin, 25 μg/mL Pepstatin, 2.0 μg/mL Aprotinin, 1 mM PMSF, pH 7.5) (1 × 10 ^7^ cells/mL). Samples were allowed to sit on ice for 15 min and the centrifuged at 2,000 x g for 5 min. The supernatant was collected and transferred to a clean test tube. The plates were prepared and assay was performed according to the manufacturer's instructions. A microplate reader was used to immediately determine the optical density of each well at 450 nm.

### Western Blotting

After three washes with cold phosphate buffered saline (PBS), cells were harvested using cell scrapers. Protein quantitation was performed using the BCA method. Equal amounts of extracted proteins were loaded on sodium dodecyl sulfate-polyacrylamide gels and electrophoresed for 1.5 h at 80 mV. Proteins were then transferred to NC membranes at room temperature for 1 h at 100 mV. Membranes were blocked with 5% non-fat milk for 1 h and incubated with indicated primary antibodies overnight at 4°C. Following this, membranes were washed five times with PBS-T over a 40-min timespan and incubated with appropriate secondary antibodies (Santa Cruz Biotechnology) for 1 h at room temperature. After 4–5 washes with PBS-T over a 40-min timespan, membranes were treated with enhanced chemiluminescence (ECL) reagents to detect the immune complexes.

### RNA Isolation and Real-Time RT-PCR

RNA isolation and real-time RT-PCR were performed as previously described (22). The miRNA isolation kit (Ambion) and TaqMan miRNA reverse transcription kit (Applied Biosystems) (Carlsbad, CA, USA) were utilized to extract and reverse transcribe miRNA, respectively. qRT-PCR analysis of miR-200a was performed with the TaqMan premix (Takara, Shiga, Japan). The primers used for reverse transcription and probes for qRT-PCR were purchased from Applied Biosystems.

### *In vitro* Migration and Invasion Assays

For the transwell migration assay, 2 × 10^4^ cells were seeded in the upper chamber of each insert (BD Biosciences, NJ, USA) with an uncoated membrane. For the invasion assay, 5 × 10^4^ cells were seeded in the upper chamber of each insert (BD Biosciences, Billerica, MA, USA) with a Matrigel-coated membrane. After incubation for 24 h at 37°C, a dye solution containing 0.1% crystal violet and 20% methanol was used to fix and stain the migrated or invaded cells. Cells were then visualized and the number of migrated/invaded cells was counted using an inverted IX71 microscope (Olympus Corp., Tokyo, Japan).

### Statistical Analysis

All data are presented as the mean ± standard error of the mean (SEM) from at least three independent experiments. Statistical analysis was performed with the Student's *t*-test or one-way analysis of variance (ANOVA) followed by Dunnett's test where appropriate. *p* < 0.05 was considered to be statistically significant.

## Results

### Activation of PDGF-B/PDGFR Promotes Cell Proliferation and Metastatic Phenotype in TNBC

**Figure 1:**
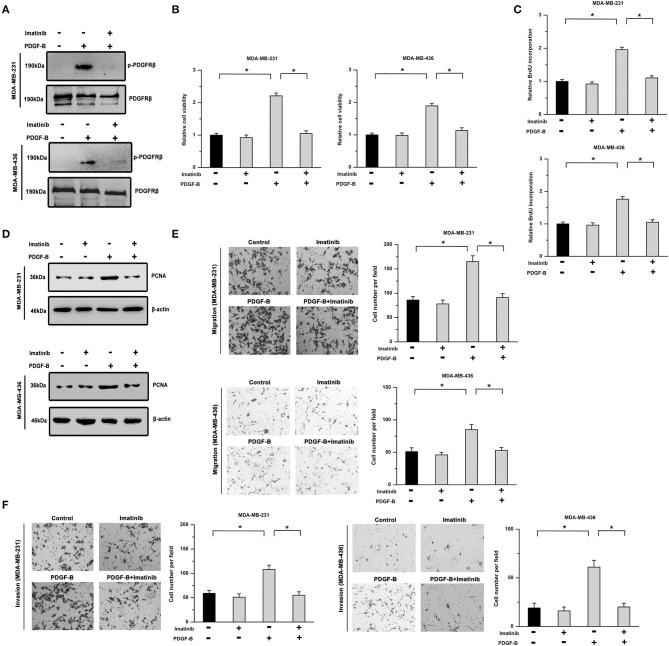
Cell proliferation and metastatic phenotype are facilitated by activation of the PDGF-B/PDGFR pathway. **(A)** PDGF-B induced the phosphorylation of PDGFRβ, which was significantly mitigated by treatment with imatinib (5 μM). **(B)** The PDGF-B (20 ng/mL)-induced increase in cell viability was inhibited by imatinib treatment in TNBC cells. **(C,D)** PDGF-B treatment promoted BrdU incorporation **(C)** and induced the expression of PCNA **(D)**. However, these effects were attenuated by PDGFR inhibition with 5 μM imatinib. **(E,F)** The promoting effects of PDGF-B on cell migration **(E)** and invasion **(F)** were mitigated by treatment with a PDGFR inhibitor (imatinib). *Indicates *P* < 0.05.

To determine the roles of PDGF-B/PDGFR in TNBC, two TNBC cell lines (MDA-MB-231 and MDA-MB-436) treated with exogenous PDGF-B (20 ng/mL) and/or imatinib (an inhibitor of PDGFR, 5 μM) were utilized. First, we examined the phosphorylation of PDGFRβ (Y1021). Our results showed that the PDGF-B-induced increase in PDGFRβ phosphorylation was significantly repressed by treatment with imatinib in TNBC cells (Figure 1A). Moreover, PDGF-B treatment increased cell viability, BrdU incorporation, and PCNA expression, while inhibition of PDGFR with imatinib abolished the effects of PDGF-B on cell proliferation (Figures 1B–D). Next, we examined the roles of PDGF-B/PDGFR in cell migration and invasion. As shown in Figures 1E,F, treatment with PDGF-B promoted the migration and invasion of TNBC cells; however, these effects were attenuated by PDGFR inhibition.

**Figure 2:**
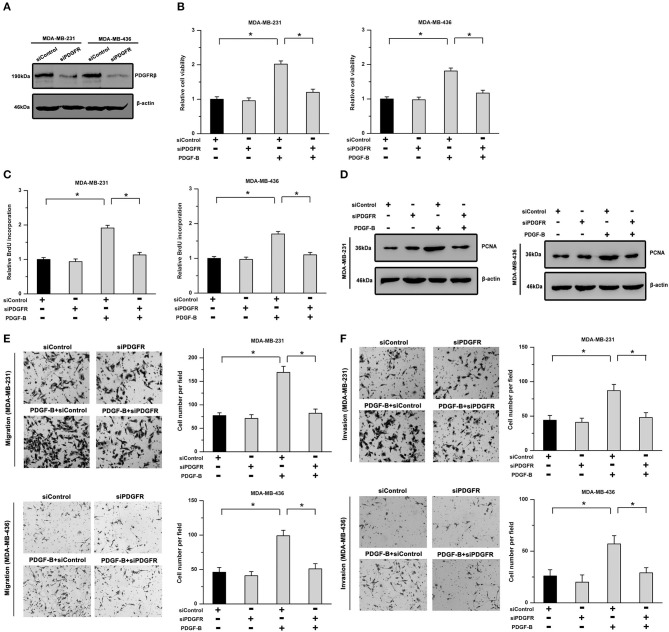
The PDGF-B-induced effects on cell proliferation and metastatic phenotype are suppressed by knockdown of PDGFR. **(A)** The knockdown efficiency of PDGFRβ in TNBC cells was validated by western blot analysis. **(B)** The PDGF-B-induced increase in cell viability was abolished by knockdown of PDGFR. **(C,D)** Treatment with PDGF-B induced BrdU incorporation **(C)** and PCNA expression **(D)**; however, these effects were attenuated by PDGFR knockdown. **(E,F)** PDGF-B-induced metastatic phenotype in TNBC cells was antagonized by reducing the expression of PDGFR. *Indicates *P* < 0.05.

Furthermore, to exclude any possible non-specific inhibition induced by imatinib, we utilized siRNA to downregulate PDGFRβ expression. As shown in Figure 2A, the knockdown efficiency was verified by western blot analysis in TNBC cells. Our results showed that the promoting effects of PDGF-B on cell viability, BrdU incorporation, and PCNA expression were significantly mitigated by knockdown of PDGFR (Figures 2B–D). Moreover, the migration and invasion abilities potentiated by PDGF-B were abrogated by PDGFR knockdown in TNBC cells (Figures 2E,F). These results indicate that activation of PDGF-B/PDGFR promotes cell proliferation and metastatic phenotype in TNBC.

### The Effects of PDGF-B on Cell Proliferation Are Mediated by SHP-2

**Figure 3:**
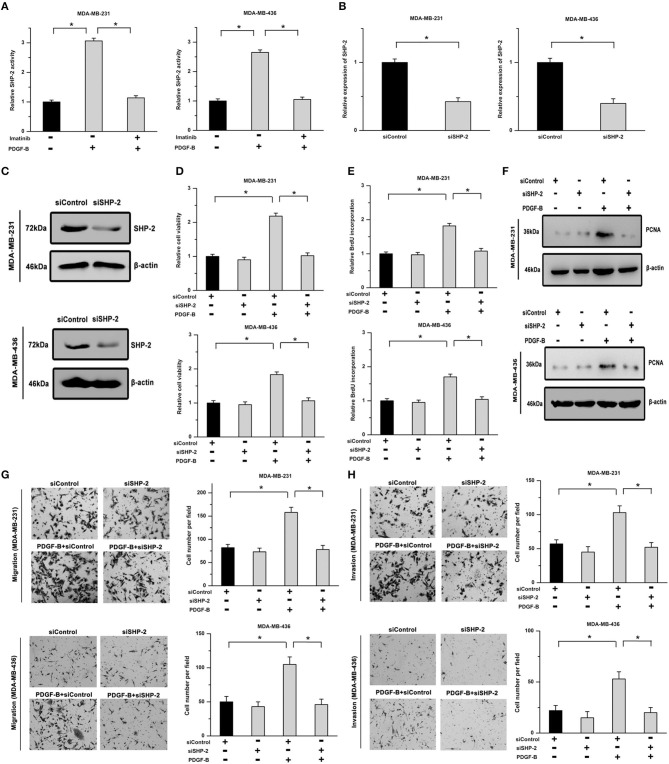
SHP-2 is involved in PDGF-B-induced promotion of TNBC cell proliferation and metastatic phenotype. **(A)** Activation of the PDGF-B/PDGFR pathway resulted in increased SHP-2 activity. **(B,C)** The expression of SHP-2 was significantly decreased by siSHP-2 transfection, as indicated by real-time RT-PCR **(B)** and western blot **(C)** analyses. **(D)** The PDGF-B-induced increase in cell growth was inhibited by knockdown of SHP-2. **(E,F)** The increase in BrdU incorporation **(E)** and PCNA expression **(F)** induced by PDGF-B treatment was mitigated by siSHP-2 transfection in TNBC cells. **(G,H)** The promoting effects of PDGF-B on cell migration **(G)** and invasion **(H)** were inhibited by SHP-2 knockdown. *Indicates *P* < 0.05.

SHP-2 participates in regulating many cellular physiological processes and acts as an important signaling molecule in PDGFR-mediated cellular functions. Thus, we examined whether SHP-2 is involved in PDGF-B-mediated cell growth. As shown in Figure 3A, the activity of SHP-2 was significantly increased by treatment with PDGF-B, but mitigated by PDGFR inhibition. To explore the roles of SHP-2 in TNBC cells, its expression was knocked down using SHP-2 siRNA (siSHP-2). The SHP-2 knockdown efficiency was confirmed by real-time PCR and western blot analyses (Figures 3B,C). Our results showed that the promoting effects of PDGF-B on cell viability, BrdU incorporation, and PCNA expression were attenuated by siSHP-2 transfection (Figures 3D–F). Additionally, knockdown of SHP-2 inhibited the PDGF-B-induced increase in cell migration and invasion (Figures 3G,H).

### SHP-2 Activated by PDGF-B Participates in Regulating TNBC Cell Proliferation via ZEB1

**Figure 4:**
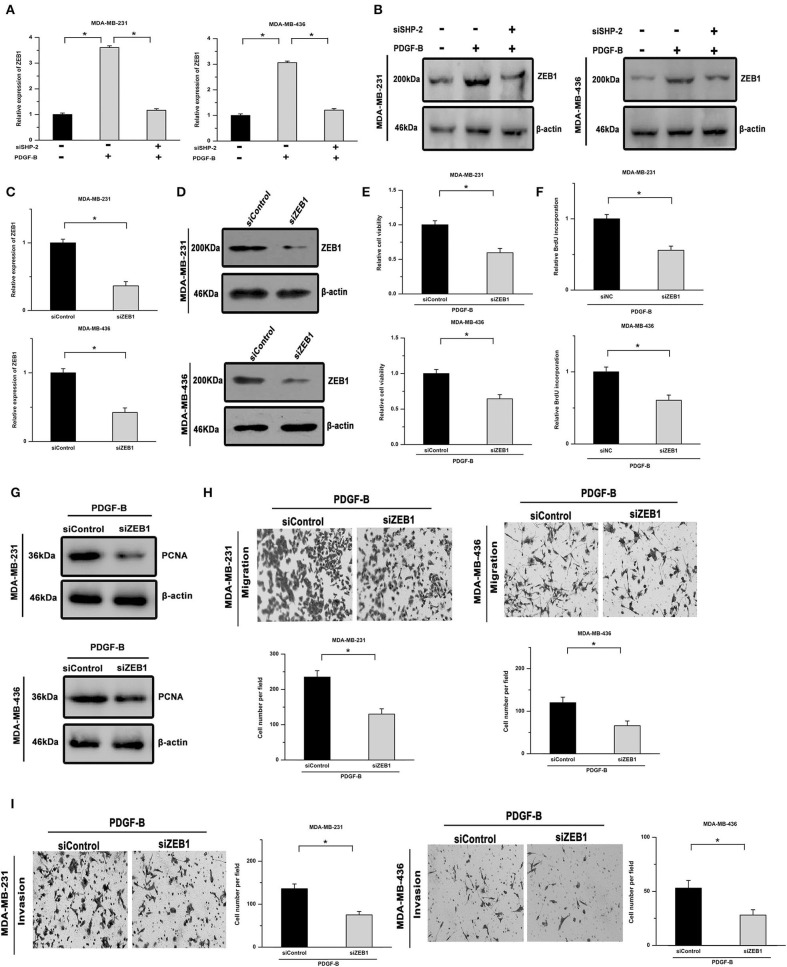
PDGF-B-mediated SHP-2 activation promotes cell proliferation by positively regulating ZEB1 in TNBC. **(A,B)** Treatment with PDGF-B increased the mRNA **(A)** and protein levels **(B)** of ZEB1 in TNBC cells, and this effect was mitigated by knockdown of SHP-2. **(C,D)** The ZEB1 knockdown efficiency was verified by real-time RT-PCR **(C)** and western blot **(D)** analyses. **(E)** The PDGF-B-induced increase in cell growth was inhibited by transfection with siZEB1. **(F,G)** The promoting effects of PDGF-B on BrdU incorporation **(F)** and PCNA expression **(G)** were abrogated by knockdown of ZEB1. **(H,I)** The promoting effects of PDGF-B on cell migration **(H)** and invasion **(I)** were antagonized by siZEB1 transfection. *Indicates *P* < 0.05.

ZEB1, an inducer of EMT, is closely related to SHP-2-mediated cellular functions and serves as a downstream effector of SHP-2. As shown in Figures 4A,B, the mRNA and protein levels of ZEB1 were increased by PDGF-B treatment in TNBC cells, while knockdown of SHP-2 suppressed this effect. To clarify the role of ZEB1 in PDGF-B-induced cell proliferation, its expression was knocked down using ZEB1 siRNA (siZEB1) (Figures 4C,D). The increase in cell viability and BrdU incorporation induced by PDGF-B treatment was abolished by siZEB1 transfection (Figures 4E,F). Moreover, knockdown of ZEB1 inhibited the PDGF-B-induced increase in PCNA expression (Figure 4G). Furthermore, after suppression of ZEB1 expression, the PDGF-B-induced increase of metastatic phenotype in TNBC cells was antagonized (Figures 4H,I). These results suggest that ZEB1 acts as an important downstream effector of PDGF-B/SHP-2 in TNBC cells.

### miR-200a Is Involved in the Regulatory Effects of PDGF-B/SHP-2 on ZEB1 in TNBC

**Figure 5:**
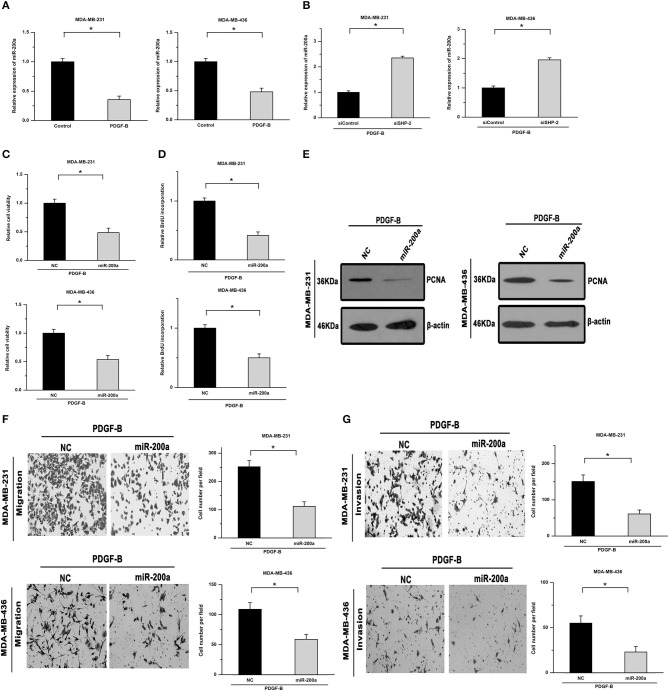
miR-200a mediates the regulatory effects of PDGF-B/SHP-2 on ZEB1. **(A)** Treatment with PDGF-B suppressed the expression of miR-200a. **(B)** The inhibitory effects of PDGF-B on miR-200a were abolished by knockdown of SHP-2. **(C)** The PDGF-B-induced increase in cell growth was mitigated by miR-200a transfection. **(D,E)** The increase in BrdU incorporation **(D)** and PCNA expression **(E)** induced by PDGF-B treatment was abrogated by transfection with miR-200a. **(F,G)** PDGF-B-induced cell migration **(F)** and invasion **(G)** were attenuated by miR-200a transfection. *Indicates *P* < 0.05.

Members of the miR-200 family, especially miR-200a, can post-transcriptionally regulate ZEB1 expression. Hence, we next examined whether miR-200a is involved in mediating the regulatory effects of PDGF-B on TNBC cell proliferation. As shown in Figures 5A,B, treatment with PDGF-B repressed the expression of miR-200a, while knockdown of SHP-2 abolished the inhibitory effects of PDGF-B on miR-200a expression. Moreover, the PDGF-B-induced increase in cell viability, BrdU incorporation, and PCNA expression was significantly mitigated by miR-200a transfection (Figures 5C–E). The cell migration and invasion abilities, which were increased by treatment with PDGF-B, were reduced by miR-200a transfection (Figures 5F,G). These results indicate that PDGF-B promotes the growth and metastatic phenotype of TNBC cells by repressing miR-200a expression.

### The Regulatory Effects of PDGF-B on miR-200a and ZEB1 Are Mediated via the Akt Pathway

**Figure 6:**
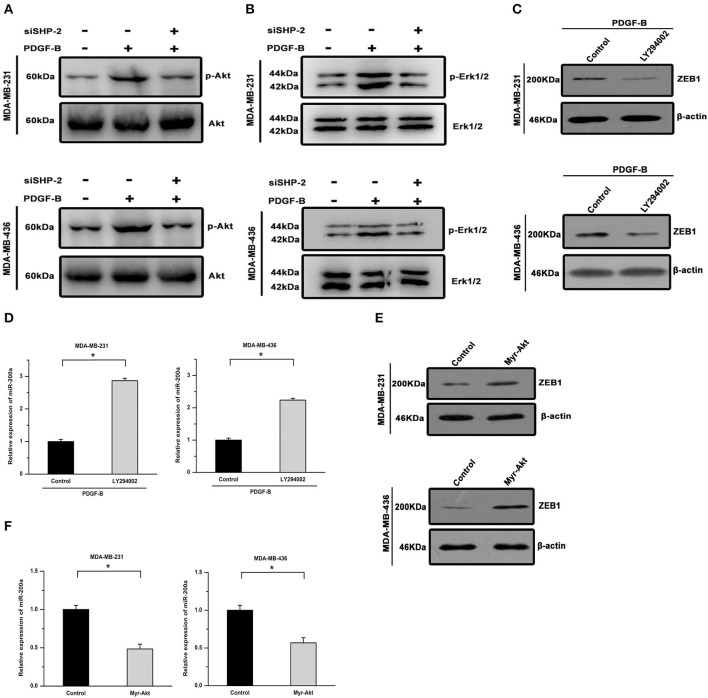
The regulatory effects of PDGF-B/SHP-2 on miR-200a and ZEB1 are mediated by the Akt pathway. **(A)** The promoting effect of PDGF-B on Akt phosphorylation was reversed by knockdown of SHP-2. **(B)** The PDGF-B-induced increase in Erk1/2 phosphorylation was mitigated by SHP-2 knockdown. **(C,D)** Treatment with PDGF-B induced ZEB1 expression **(C)** and repressed miR-200a expression **(D)**; however, these effects were antagonized by inhibition of the Akt pathway with LY294002. **(E,F)** Activation of Akt via overexpression of the Myr-Akt mutant promoted ZEB1 expression **(E)** and repressed miR-200a expression **(F)** in TNBC cells. *Indicates *P* < 0.05.

We further investigated the signal transduction pathway connecting PDGF-B/SHP-2 and miR-200a/ZEB1. The Akt pathway has been shown to play important roles in physiological processes regulated by either PDGF-B or SHP-2. Hence, we investigated its roles in the regulation of miR-200a/ZEB1 by PDGF-B. As shown in Figure 6A, PDGF-B treatment increased Akt phosphorylation, but this effect was attenuated by SHP-2 knockdown. Similarly, PDGF-B-induced activation of Erk was mitigated by SHP-2 knockdown (Figure 6B). Moreover, the regulatory effects of PDGF-B on the expression of ZEB1 and miR-200a were antagonized by treatment with 10 μM LY294002 (an Akt pathway inhibitor) (Figures 6C,D). Conversely, overexpression of a constitutively activated Akt (Myr-Akt) mutant induced ZEB1 expression and repressed miR-200a expression in TNBC cells (Figures 6E,F). These results imply that the by PDGF-B-induced regulation of miR-200a/ZEB1 is mediated via the SHP-2/Akt pathway.

## Discussion

Currently, TNBC, which is a more aggressive and more highly metastatic malignant tumor than other breast tumors, still lacks an effective molecular therapy. This is majorly because the specific oncogenic drivers of TNBC progression and underlying mechanism remain largely unknown. The results of the present study demonstrated the critical role of PDGF-B/PDGFR in the proliferation and metastatic phenotype of TNBC cells. In this mechanistic study, we showed that PDGF-B-mediated SHP-2 activation promotes cell proliferation and metastatic phenotype by regulating miR-200a and ZEB1 expression. These findings reveal important regulatory molecules involved in TNBC development that might serve as potential therapeutic targets.

Next, we showed that PDGF-B/PDGFR facilitate cell proliferation and invasion by upregulating the activity of SHP-2. The roles of PDGF and PDGFR have been demonstrated in various types of tumors, including breast cancer. However, the mechanism of action remains to be elucidated. SHP-2, implicated in the activation of Ras-MAPK signaling, has been demonstrated to be activated by PDGF/PDGFR signaling (23, 24). Activation of SHP-2 has been reported in certain types of cancers, such as hematological malignancies and glioblastoma (25). A previous study showed that PDGF-stimulated glioma cell migration is mitigated by a dominant negative SHP-2 mutation (26), but whether SHP-2 is involved in PDGF-regulated progression of TNBC remains controversial. Here, we found that treatment with PDGF-B enhanced the activity of SHP-2 in TNBC cells. In addition, knockdown of SHP-2 antagonized the promoting effects of PDGF-B on cell proliferation and invasion. These results indicate that SHP-2 plays important roles in the PDGF-B/PDGFR-stimulated growth and metastatic phenotype in TNBC cells.

In this study, our findings further revealed that PDGF-B-mediated SHP-2 activation induced ZEB1 expression and repressed miR-200a expression. ZEB1, a well-known regulator of EMT, has been proven to participate in the modulation of cell growth and metastasis in various types of cancers. Knockdown of ZEB1 via RNA interference was shown to inhibit cell growth and colony formation and induce apoptosis in NSCLC cells (14). Targeting of ZEB1 is considered a potential therapeutic strategy for mitigating cancer cell migration and invasion. In glioblastoma cells, the ZEB1 pathway is closely related to tumor initiation, invasion, and chemoresistance (27). Furthermore, miR-200a, a member of the miR-200 family, acts as a negative regulator of ZEB1. miR-200a has been reported to inhibit tumor cell EMT and metastasis by suppressing the expression of ZEB1 in cancer cells (28, 29). Here, we found that PDGF-B treatment induced ZEB1 expression and inhibited the expression of miR-200a; however, these effects were attenuated by knockdown of SHP-2. Moreover, the promoting effects of PDGF-B on cell proliferation and metastatic phenotype were mitigated by transfection with either siZEB1 or miR-200a. These results indicate that PDGF-B-mediated SHP-2 activation regulates the progression of TNBC via the miR-200a-ZEB1 loop.

To date, several SHP2 inhibitors have been developed and investigated. However, they have not been utilized for clinical application due to the complex regulatory mechanisms of SHP2 upstream and downstream. Many pathways participate in SHP2 regulation and are potential targets of SHP2 inhibitors. SHP099, the first selective and orally bioavailable allosteric PTP inhibitor specific to SHP2, targets RTK-driven tumors (30) and can inhibit the growth of resistant PDCs in combination with ALK TKI (31). RMC-4550, a small molecular allosteric inhibitor, is effective in tumors with RAS-GTP-dependent oncogenic BRAF, NF1 loss, or nucleotide-cycling oncogenic RAS (32). Moreover, SHP2 inhibitors have been shown to prevent adaptive resistance to MEK inhibitors (33), and dual SHP2/MEK inhibition is a clinically viable targeted therapy approach for KRAS-mutant tumors (34). In this study, our results indicated that SHP2 is essential for PDGF-B/PDGFR-mediated regulation of miR-200a/ZEB1 in TNBC cells *in vitro*, implying that SHP2 may be a novel therapeutic target for inhibiting the progression of TNBC. However, the application of SHP2 inhibitors in TNBC treatment requires further study and effective methods to reverse the resistance and side-effects of inhibitors need to be developed.

This study has certain limitations. Although we demonstrated the important role of SHP-2/ZEB1 in PDGF-induced TNBC cell proliferation *in vitro*, the underlying regulatory mechanisms need to be validated *in vivo* in future studies. Furthermore, due to the existence of bypass activation, other factors may also be involved in the PDGF-SHP2-miR200/ZEB1 pathway in TNBC, which requires further examination.

In summary, our results show that PDGF-B/PDGFR promote cell growth and invasion by enhancing the activity of SHP-2. Moreover, PDGF-B/SHP-2 regulates the progression of TNBC via the miR-200a-ZEB1 loop. Hence, our findings reveal a critical regulatory role of PDGF-B/PDGFR in TNBC development and the underlying molecular mechanism, possibly providing a new therapeutic strategy for TNBC.

## Data Availability Statement

All datasets generated for this study are included in the article/supplementary material.

## Author Contributions

JL, JM, and LZha conceived and coordinated the study, drafted and revised the manuscript. CY and JP performed the cell culture and some cellular experiments. LZha, LZho, and YL performed the molecular experiments. LZha, WY, YJ, and SX analyzed the data. All authors read and approved the final manuscript.

## Conflict of Interest

The authors declare that the research was conducted in the absence of any commercial or financial relationships that could be construed as a potential conflict of interest.
